# Benign Smooth Muscle Tumors (Leiomyomas) of Deep Somatic Soft Tissue

**DOI:** 10.1155/2018/2071394

**Published:** 2018-09-09

**Authors:** Aoife J. McCarthy, Runjan Chetty

**Affiliations:** Department of Anatomical Pathology, Laboratory Medicine Program, University Health Network and University of Toronto, Toronto, ON, Canada

## Abstract

Leiomyomas of deep soft tissue are extremely rare and should only be diagnosed following adherence to stringent histological criteria, namely, the absence of nuclear atypia and of coagulative tumor necrosis. Whether extremely low counts of, or even any, mitotic activity are acceptable when making a diagnosis of leiomyoma in deep soft tissue sites is controversial. The morphology and immunophenotype of smooth muscle tumors in deep soft tissue are similar to their counterparts irrespective of topography. It is interesting to note that leiomyomas of deep soft tissue (extremity and retroperitoneum) are often hyalinized/sclerosed and calcified. However, the prediction of their behavior and correct codification is dependent on thorough, meticulous search for mitoses and necrosis. Leiomyomas of deep soft tissue in the extremity should be devoid of mitoses and “significant” cytological atypia. An occasional larger, slightly pleomorphic cell in the midst of bland spindle cells, can be regarded as insignificant atypia. If any mitotic activity and several atypical cells are encountered in smooth muscle tumors of deep soft tissue of the extremity, it would be prudent to invoke the appellation of smooth muscle tumor of uncertain malignant potential and advocate wide local excision and follow-up.

## 1. Introduction

In the past, the existence of leiomyomas of deep soft tissue was considered controversial. The prevailing view was that most, and perhaps all, smooth muscle tumors of deep soft tissue were malignant, although this was based on studies of smooth muscle tumors demonstrating both mitotic activity and cytological atypia. To date, there have been very few studies of well-differentiated smooth muscle tumors of deep soft tissue that display an absence of mitotic activity and cytological atypia. It is now thought that, although they are exceptionally rare, deep soft tissue leiomyomas do in fact exist [1–8] and can occur in somatic soft tissue or in the retroperitoneum and abdominal cavity.

In the literature, leiomyoma of deep soft tissue is scarcely mentioned, hence much of our knowledge regarding this entity is based on case reports and exiguous small case series [9, 10].

The focus of this succinct review is on leiomyomas of deep somatic soft tissue.

## 2. Clinical Features

Leiomyomas of deep somatic soft tissue occur primarily in middle-aged adults, with a mean age of 37 years, and with equal distribution among the genders [10]. Deep somatic soft tissue of the lower extremity, usually the thigh, is the most common site, followed by the upper extremity and the trunk [10]. Many of these lesions are calcified, likely resulting from regressive changes, and can be detected radiographically [4, 5, 11–13]. The radiological appearances are usually nonspecific, and differential diagnoses include calcifying schwannoma, chondroma, myositis ossificans, tumoral calcinosis, chondrosarcoma, and synovial sarcoma [13]. Thus, wide local excision is required for definitive classification.

## 3. Gross Appearance

Grossly, the majority of deep somatic soft tissue leiomyomas are circumscribed masses, with a tan-grey to white cut surface, and a mean size of 5.4 cm. Degenerative mucoid-cystic change is occasionally present [10].

## 4. Histological Features

**Figure 1:**
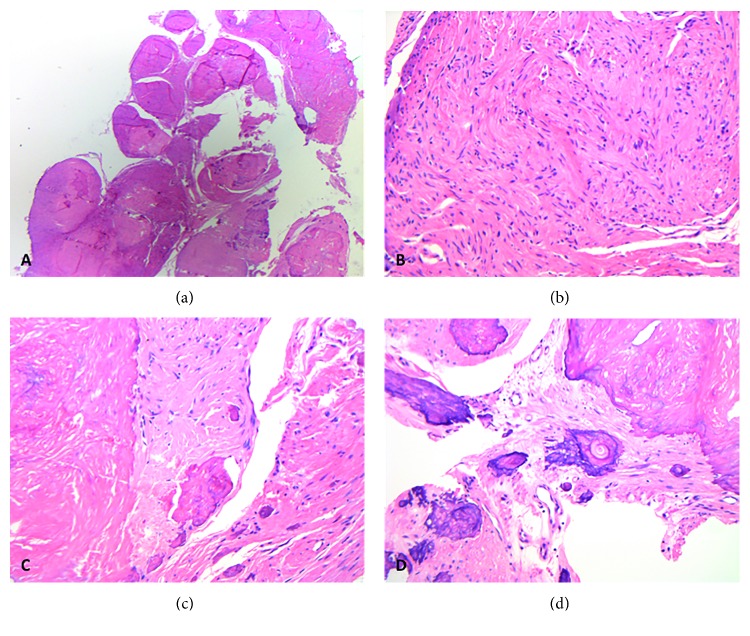
Benign smooth muscle tumor (leiomyoma) of deep somatic soft tissue of the extremity: histologically, an unencapsulated, well-circumscribed, multinodular, low cellularity spindle cell lesion is usually seen (a). The lesional cells are characteristically bland, cigar-shaped spindle cells, with eosinophilic cytoplasm and indistinct cell borders. There is no evidence of mitotic activity, nuclear pleomorphism, or coagulative tumor necrosis (b). Hyalinization and calcification may be present (c). Osseous metaplasia was also seen in this particular case (d).

Microscopically, deep soft tissue benign smooth muscle tumors are often well-circumscribed, unencapsulated, occasionally multinodular, paucicellular spindle cell lesions (Figure 1(a)). The lesional cells are bland, cigar-shaped spindle cells, with indistinct cell borders and abundant light to deeply eosinophilic cytoplasm arranged in oblique or perpendicular fascicles (Figure 1(b)). Stromal hyalinization occurs in the majority of cases and ranges from mild perivascular hyalinization to dense hyalinization (Figure 1(c)). Prominent stromal calcification is present in many of these lesions [10], and osseous metaplasia can also be encountered (Figure 1(d)). There is no evidence of mitotic activity, and nuclear pleomorphism and coagulative tumor necrosis are absent (Table 1).

While an important diagnostic tenet of leiomyomas of deep somatic soft tissue is that nuclear atypia is lacking in the majority, focal minimal atypia has been described in a few cases [10]. Mitoses are very rarely identified and typically do not number more than 1 mitosis per 50 high power fields (HPF). Atypical mitotic figures are not present [10]. It is essential to search assiduously for the presence of “coagulative tumor necrosis,” and this must be distinguished from “hyaline necrosis.” Hyaline necrosis is characterized by necrosis which is separated from viable tumor by a zone of connective tissue that varies from granulation tissue to hyalinized fibrous tissue, depending on the age of the necrosis [14]. In contrast, coagulative tumor necrosis is defined by an abrupt transition from viable cells to necrotic cells without the interposed zone of granulation or fibrous tissue of infarct-type necrosis [14]. The presence of coagulative tumor necrosis is not compatible with a diagnosis of benign smooth muscle tumor or leiomyoma in deep somatic soft tissue.

In 2001, Billings and colleagues analyzed the clinicopathological features of 13 highly differentiated smooth muscle tumors/leiomyomas of deep somatic soft tissue (as well as, but separately to, those in the retroperitoneum and abdominal cavity) to determine if it is indeed possible to define a biologically benign group of smooth muscle tumors at this site by using stringent criteria [10]. Nuclear atypia was lacking in the majority of leiomyomas of deep somatic soft tissue in their series, although minimal atypia of a focal nature was present in occasional cases. 11 of 13 cases had a mitotic index of ≤1 mitosis/50 HPF, and all cases had a mitotic count ≤4 mitoses/50 HPF. The authors stress that, in assessing smooth muscle tumors of deep soft tissue, it is essential to distinguish coagulative tumor necrosis from hyaline necrosis, and none of their cases contained the former [10].

Billings et al. cautioned that their study encountered three leiomyomas of somatic soft tissue with low levels of mitotic activity (1–4 mitoses per 50 HPF), all of which occurred in young individuals (ages 6, 12, and 21 years, resp.) [10]. Follow-up was available for two of these patients, ranging from 4 to 38 months only, and both patients had no adverse events. Although it is well established that mitotic activity of *bona fide* leiomyosarcomas of deep somatic soft tissue is usually higher than that in these 3 cases [15], given the absence of long-term follow-up for these patients, it is reasonable to be cautious in classifying these lesions as outright benign leiomyomas [10]. Thus, the authors concluded that highly/well-differentiated smooth muscle tumors of deep somatic soft tissue with minimal/low mitotic activity should be designated as smooth muscle tumors of uncertain malignant potential, until proven otherwise [10].

## 5. Immunohistochemistry and Ancillary Tests

**Figure 2:**
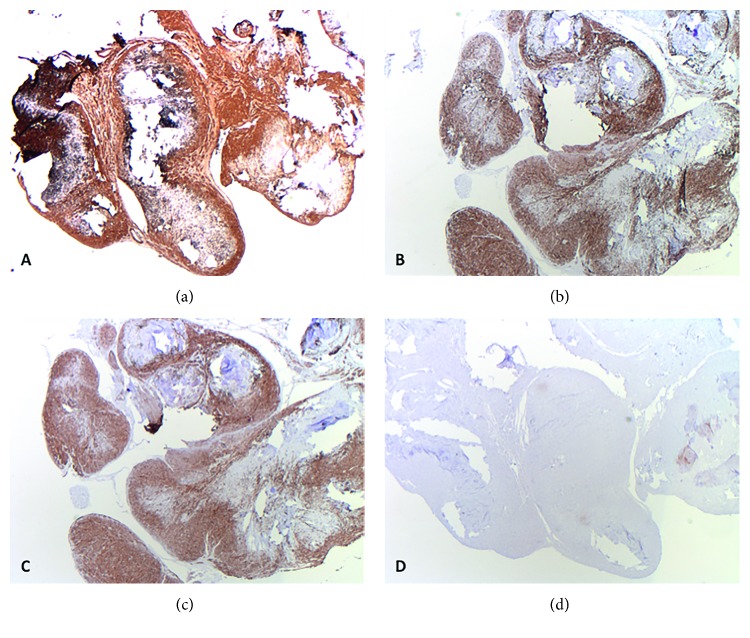
Immunohistochemistry of a benign smooth muscle tumor (leiomyoma) of deep somatic soft tissue of the extremity: the lesional cells are strongly and diffusely positive for smooth muscle actin (a), desmin (b), and caldesmon (c) and negative for S100 (d) and SOX-10 (not shown).

Leiomyomas of deep somatic soft tissue are positive for smooth muscle actin (SMA), desmin, and caldesmon (Figure 2) and expectedly are negative for a range of markers including cytokeratins, S100 protein, SOX-10, and CD34.

Genetics of deep soft tissue smooth muscle tumors are as yet poorly understood, and it remains to be seen if genes such as *MED12* are indeed mutated in this particular group of benign smooth muscle tumors. Therefore, such diagnostic testing is not yet generally applicable in this histogenetic group.

## 6. Differential Diagnosis

Differential diagnoses of benign smooth muscle tumors (leiomyomas) of deep somatic soft tissue include both benign and malignant entities.

### 6.1. Angioleiomyoma

Angioleiomyoma is the most common peripheral soft tissue leiomyoma, typically occurring in the subcutis of extremities, particularly the lower leg, but also in the trunk wall and less commonly in the head and neck region. It usually forms a small 1-2 cm well-circumscribed, homogeneous rubbery nodule, and it is clinically notable for often being painful [16]. Histologically, angioleiomyomas are composed of eosinophilic smooth muscle cells intimately associated with a vein wall, forming swirling congeries of spindle cells closely apposed to the vessel wall. The recognized variants are solid (very small lumens), venous (medium-size lumens), and cavernous (large lumens and thin smooth muscle elements in between) [17]. Focal calcification can be present [16]. Angioleiomyomas have an identical immunophenotype to leiomyomas of deep soft tissue, being positive for SMA and desmin and negative for S100 protein [17]. Some examples of angioleiomyoma are positive for CD34 [17]. Thus, the identification of the association with a vein wall is key in distinguishing angioleiomyoma from leiomyomas of deep soft tissue.

### 6.2. Epstein–Barr Virus- (EBV-) Associated Smooth Muscle Tumor

EBV-associated smooth muscle tumor is a rare subset of smooth muscle tumor that occurs in immunosuppressed individuals. It is most common in patients with human immunodeficiency virus (HIV), many of whom are young, including children. EBV-associated smooth muscle tumors also occur in solid organ transplant recipients (liver, kidney, and heart), who are usually middle-aged adults, and rarely in patients with a congenital immunodeficiency disorder [18]. It can occur in peripheral soft tissue, as well as in the intracranial space, or visceral sites [16, 19]. Histologically, EBV-associated smooth muscle tumors consist of a fascicular arrangement of relatively well-differentiated smooth muscle cells with brightly eosinophilic cytoplasm and elongated, blunt-ended nuclei exhibiting variable atypia [18]. Necrosis may be present, and variable mitotic activity can be seen [18]. The two important defining and unique features are the presence of variable numbers of intratumoral T-lymphocytes and of so-called primitive round cell areas arising gradually or abruptly from the well-differentiated smooth muscle cells [19]. They are no longer subdivided morphologically into EBV-associated leiomyomas and leiomyosarcomas, but all are categorized under the umbrella term of EBV-associated smooth muscle tumors. These tumors are SMA positive but are often desmin negative. A prerequisite for the distinction of EBV-associated smooth muscle tumors from leiomyomas of deep soft tissue is the demonstration of EBV-RNA by in situ hybridization, which highlights the tumor cell nuclei of EBV-associated smooth muscle tumors. It is essential to correctly identify this category of smooth muscle tumors, as, although they are usually indolent, they can be attended by a somewhat unpredictable behavior.

### 6.3. Leiomyosarcoma

Smooth muscle tumors in soft tissue that contain both nuclear atypia and mitotic activity are generally designated leiomyosarcomas to denote their malignant (metastatic) potential [16]. Histologically, leiomyosarcomas are composed of irregularly intersecting fascicles of spindled cells with variably eosinophilic cytoplasm and variable mitotic activity [16]. Nuclei are typically blunt-ended, cigar-shaped, and these histological features are sufficient for the recognition of most leiomyosarcomas [16]. A great majority of soft tissue leiomyosarcomas are of intermediate or high grade, and low-grade tumors are rare. Focal pleomorphism is common even in low-grade tumors with low mitotic activity. Immunohistochemically, leiomyosarcomas are almost invariably strongly positive for SMA, and desmin is usually positive (70–80%), along with positivity for heavy-caldesmon and smooth muscle myosin [16].

### 6.4. Other Spindle Cell Neoplasms

These include nerve sheath tumors, fibrohistiocytic spindle cell lesions, and sundry spindle cell lesions with characteristic immunophenotypic profiles.

## 7. Prognosis

The small number of cases with limited follow-up period reported in the literature precludes definitive conclusions regarding the significance of low mitotic rates in smooth muscle tumors of deep somatic soft tissue.

In the study by Billings and colleagues, clinical follow-up information was available for 11 of 13 of their patients, with a mean follow-up period of 58.7 months [10]. Although 5 of 13 cases had positive resection margins, tumor recurrences or metastases were not documented [10].

However, recurrence has been reported in a smooth muscle tumor of somatic deep soft tissue with a mitotic activity of 1 mitosis per 50 HPF [20]. Fletcher et al. reported a thoroughly sampled case displaying a bland, smooth muscle lesion from the left ischiorectal fossa of a 42-year-old woman [20]. There was no evidence of cytological atypia or coagulative tumor necrosis, and there was 1 mitosis per 50 HPF. However, 16 months after resection, this lesion recurred and was now more cellular, with up to 4 mitoses per 10 HPF. This prompted the authors to conclude that any mitosis in smooth muscle tumors of subcutaneous or deep soft tissue should indicate potential malignancy, requiring wide local excision and careful follow-up [20]. This sentiment was echoed by Billings and colleagues, who suggested, as outlined previously, that well-differentiated smooth muscle tumors of deep somatic soft tissue with minimal/low mitotic activity should be considered tumors of uncertain malignant potential [10].

## 8. Management/Treatment

These lesions are managed by wide local excision. Although 5 of 13 cases reported by Billings et al. had positive resection margins, tumor recurrences or metastases were not documented [10]. The most appropriate follow-up for highly differentiated smooth muscle tumors of deep somatic soft tissue with minimal/low mitotic activity (tumors of uncertain malignant potential) has yet to be established.

## 9. Benign Smooth Muscle Tumors (Leiomyomas) of the Pelvic Retroperitoneum

As alluded to previously, leiomyomas of deep soft tissue segregate into two distinct clinicopathological groups, one group occurring in deep somatic soft tissue (the topic of this review) and the second occurring primarily in women in the pelvic retroperitoneum. For completeness, benign smooth muscle tumors (leiomyomas) of the pelvic retroperitoneum will be discussed briefly (Table 2).

Retroperitoneal leiomyomas occur preferentially in women during the perimenopausal period, usually in the pelvic retroperitoneum [21], and the lesions may be multiple [10, 22]. The histological appearance is per that described above for deep somatic soft tissue leiomyomas. However, unlike somatic soft tissue leiomyomas, 20% of retroperitoneal/abdominal leiomyomas display low levels of mitotic activity (1–5 mitoses per 50 HPF) [21]. Despite this feature, less than 10% of lesions recur, and none have metastasized in follow-up periods averaging from 42 to 142 months [10, 20]. These tumors are commonly positive for estrogen receptor and progesterone receptor proteins [21].

Similar to uterine leiomyomas [23, 24], mutations in exon 2 of the *MED12* gene have been reported in extrauterine leiomyomas, including at pelvic/retroperitoneal sites [25, 26]. Recently, Panagopoulos and colleagues presented the results of eight leiomyomas of deep soft tissue (all were located in the retroperitoneum or in the abdominal/pelvic cavity) that were genetically analyzed [27]. Three tumors carried rearrangements of the long arm of chromosome 12 (the target gene of this 12q aberration was *HMGA2*), three others had 8q rearrangements (the target gene of the 8q aberration was *PLAG1*), one tumor had deletion of the long arm of chromosome 7, del(7) (q22), and one had aberrations of chromosome bands 3q21∼23 and 11q21∼22 [27]. All eight leiomyomas of deep soft tissue expressed *MED12* but none of them had mutation in exon 2 of that gene [27]. Thus, it seems that smooth muscle tumors of deep somatic soft tissue are subject to some of the same mutational changes as those of their uterine counterparts and that genetic heterogeneity is a feature of these tumors. However, further interrogation is required.

The striking female predilection of this type of leiomyoma suggests that they arise from hormonally sensitive smooth muscle and are functionally similar to uterine leiomyomas [21]. The location of these lesions and the fact that many women with these lesions had hysterectomies years previously suggest that these are not simply detached or parasitic uterine leiomyomas [10]. It is thought that, like uterine leiomyomas, mitotic activity probably does not signify malignancy in the absence of other adverse histological parameters, such as cytological atypia and coagulative necrosis [21].

## 10. Conclusion

The diagnosis of leiomyoma in deep somatic soft tissue sites should be based on stringent histological criteria, specifically extremely low levels of, or even perhaps absence of, mitotic activity and the absence of nuclear atypia and coagulative tumor necrosis [10]. Leiomyomas of deep somatic soft tissue diagnosed as such are expected to have an excellent outcome. It is, however, prudent to remember that these lesions are rare and are far outnumbered by their malignant counterpart; therefore, these lesions must be thoroughly sampled/all embedded and closely evaluated for the presence of cytological atypia, mitotic activity, and necrosis. The presence of low mitotic activity in otherwise differentiated smooth muscle tumors of deep somatic soft tissue without coagulative tumor necrosis should prompt a diagnosis of a smooth muscle tumor of uncertain malignant potential.

## Figures and Tables

**Table 1:** Summary of case series reporting the clinicopathological features of leiomyomas of deep somatic soft tissue.

	Authors; year of publication	
	Billings et al.; 2001 [10]	Kilpatrick et al.; 1994 [9]
Clinical feature		
Age	Middle-aged adults (median, 44 years; range, 12–55 years)	Median, 35 years; range 14–62 years
Gender	M = F	M = F
Anatomical site	Deep somatic soft tissue of the lower extremity, upper extremity, trunk, axilla, and back	Extremities was the most common site

Histological feature		
Architecture	Well-marginated; fascicular architecture	Well-defined circumscription; fascicular architecture
Lesional cells	Mature-appearing smooth muscle cells with abundant eosinophilic cytoplasm	Spindle cells with mostly uniform, round-ended, elongated nuclei, and tapering eosinophilic cytoplasm
Nuclear atypia	Absent; minimal atypia of a focal nature acceptable	Absent
Mitotic count	Absent or very low (≤4 mitoses/50 HPF and ≤1 mitosis/50 HPF in the majority)^*∗*^	≤1 mitosis/50 HPF
Atypical mitotic figures	Absent	Not described
Coagulative tumor necrosis	Absent	Absent
Other findings	Stromal hyalinization (majority of cases); prominent stromal calcification (some cases); degenerative nuclear changes	Dystrophic calcification (some cases); degenerative nuclear changes (some cases)

IHC		
ER and PR	Negative	Not described

Authors' conclusion	SMTs of somatic soft tissue that lack atypia, necrosis, and mitotic activity can with reasonable confidence be labeled leiomyomas	The presence of any mitoses in SMTs of subcutaneous or deep soft tissue should be regarded as indicative of potential malignancy; WLE and careful F/U advised

M, male; F, female; HPF, high power fields; IHC, immunohistochemistry; ER, estrogen receptor protein; PR, progesterone receptor protein; SMTs, smooth muscle tumors; WLE, wide local excision; F/U, follow-up. ^*∗*^The authors concluded that a smooth muscle tumor of somatic soft tissue with 1–5 mitoses/50 HPF should be regarded as “leiomyoma of uncertain malignant potential.”

**Table 2:** Summary of a case series reporting the clinicopathological features of leiomyomas of retroperitoneum.

	Authors; year of publication
	Billings et al.; 2001 [10]
Clinical feature	
Age	Often of perimenopausal age (median, 44 years; range 16–72 years)
Gender	F >> M
Anatomical site	Primarily in the retroperitoneum; also in the mesentery/omentum

Histological feature	
Architecture	Intersecting fascicles
Lesional cells	Mature smooth muscle cells, with bland, blunt-ended, or slightly tapered nuclei
Nuclear atypia	Absent; very focal, minimal atypia acceptable
Coagulative tumor cell necrosis	Absent
Mitotic activity	Low (mean, 1 mitosis/50 HPF; range, <1–10 mitoses/50 HPF)^*∗*^
Atypical mitotic figures	Absent
Other findings	Cystic and degenerative changes (more frequent than in counterpart in deep somatic soft tissue)

IHC	
ER and PR	Positive

M, male; F, female; HPF, high power fields; IHC, immunohistochemistry; ER, estrogen receptor protein; PR, progesterone receptor protein. ^*∗*^The authors concluded that a smooth muscle tumor of retroperitoneum with >10 mitoses/50 HPF should be regarded as “smooth muscle tumors of uncertain malignant potential.”
